# Revision of failed hip resurfacing to total hip arthroplasty rapidly relieves pain and improves function in the early post operative period

**DOI:** 10.1186/1749-799X-5-88

**Published:** 2010-11-29

**Authors:** Nemandra A Sandiford, Sarah K Muirhead-Allwood, John A Skinner

**Affiliations:** 1 The London Hip Unit, 4thFloor, 30 Devonshire Street, London, UK, W1G 6PU; 2 The Royal National Orthopaedic Hospital, Stanmore, Middlesex, UK, HA7 4LP

## Abstract

We reviewed the results of 25 consecutive patients who underwent revision of a hip resurfacing prosthesis to a total hip replacement. Revisions were performed for recurrent pain and effusion, infection and proximal femoral fractures. Both components were revised in 20 cases.

There were 12 male and 13 female patients with average time to revision of 34.4 and 26.4 months respectively. The mean follow up period was 12.7 months (3 to 31). All patients reported relief of pain and excellent satisfaction scores. Two patients experienced stiffness up to three months post operatively.

Pre operative Oxford, Harris and WOMAC hip scores were 39.1, 36.4 and 52.2 respectively. Mean post operative scores at last follow up were 17.4, 89.8 and 6.1 respectively (p < 0.001 for each score). These results show that conversion of hip resurfacing to total hip arthroplasty has high satisfaction rates. These results compare favourably with those for revision total hip arthroplasty.

## Introduction

Metal on Metal (MoM) hip resurfacing has become increasingly popular over the last decade. Data from the United Kingdom (UK) National Joint Registry [1] suggest that while hip resurfacing (HR) procedures account for approximately 10% of all hip arthroplasty procedures in the UK annually, the actual number of hip resurfacings performed is steadily increasing from 2,338 in 2004 to 5,596 in 2007 [1]. The proposed benefits of HR compared to total hip replacement include femoral bone preservation, increased stability, improved proprioception of the hip joint and technically less demanding conversion to a total hip replacement if necessary, particularly on the femoral side. This is most relevant to young, active patients.

While early results of Metal on Metal hip resurfacing have been promising, complications have been reported which require revision. These include femoral neck fractures [2] and recurrent pain and effusions thought to be related to an aseptic lymphocytic vasculitis associated lesion (ALVAL) syndrome [3]. Large destructive lesions (pseudo tumors) have also been reported which lead to soft tissue loss around the hip joint[4]. While it may be relatively straightforward to revise a hip resurfacing to a total hip replacement, the results of this procedure are unknown. If there is a complication rate of a less invasive procedure (hip resurfacing versus total hip replacement) then one needs to know the functional outcome of the revision procedure when considering it in young, active, high demand patients.

This prospective study analyses the early functional outcome of a cohort of patients who underwent conversion of a hip resurfacing to a total hip replacement. We examine the population undergoing revision and the indications for revision. Parameters examined were the Oxford, Harris and Western Ontario McMaster (WOMAC) hip scores, relief of pain and patient satisfaction.

## Patients and Methods

Twenty five consecutive patients underwent revision of resurfacing components to total hip arthroplasty in our unit between 2006 and 2008. This cohort included 12 male and 13 female patients. Twenty patients had revision of both components while the remaining five underwent revision of the femoral component only. Pre and post operative Oxford, Harris and WOMAC hip scores as well as the University of California Los Angeles (UCLA) activity scores (Table 1) were collected. Other data including gender, age, time to failure of the original implant and reasons for failure were recorded (Table 2, 3). All hip scores were collected prospectively.

**Table 1:** Modified University of California Los Angeles (UCLA) activity scale

Category	Activity level
1	Inactive: Wholly inactive. Dependent on others. Cannot leave residence
2	Mostly inactive: Restricted to minimum activities of daily living.
3	Mild activity: Sometimes participates in mild activities such as walking, limited housework and shopping.
4	Regularly participates in mild activities. *Sedentary occupational work*.
5	Moderate activity: Sometimes in moderate activities such as swimming and can do unlimited housework or shopping.
6	Regularly participates in moderate activities. *Light occupational work*
7	Active Regularly participates in active events such as bicycling, *aqua-aerobics. Gardening or working out in the gym once or twice a week*.
8	Very active: Regularly participates in very active events such as bowling, golf. *Riding, hunting, aerobics. Gardening or working out in the gym three times per week or more. Moderately heavy occupational work. Farming*.
9	Impact sports: Sometimes participates in impact sports such as *running*, jogging, tennis, *cricket, baseball, rugby, football, hockey, racquet sports, judo, karate and other martial arts*, skiing, acrobatics, ballet dancing, backpacking and *mountaineering*. *Heavy occupational work*.
10	Regularly participates in impact sports as described above

**Table 2:** Patient Demographics

	Males	Females
Number of patients	13	12
Mean Age/years	62.2 (56-72)	58.5 (41 - 65)
Time to revision (months)	34.4 (4-65)	26.4 (7-60)
Infections	1	1
Femoral neck fractures (due to falls)	2	0
Femoral component size	49 (46-54)	43 (38-50)
Retained acetabular components	4	1

**Table 3:** Indications for revision

Diagnosis	Number of patients
Infection	2
Groin pain	6
Unexplained pain after sport	2
Pain with clicking	2
Pain with effusion	10
Dislocation	1
Femoral neck fracture secondary to fall	2

All revision procedures were performed by a single surgeon (SM-A) via a posterior approach using uncemented components. In all cases where infection was suspected, capsular tissue as well as culture swabs of both components and samples of any effusions were sent for microbiological analysis. Statistical analysis was carried out using the unpaired student's t-test (Graph pad Prism software, California, USA)

## Pre operative planning

Pre operative investigations included standard anteroposterior and lateral x-rays of the pelvis and affected hip respectively. Suspected acetabular defects were further investigated by computerized tomography (CT) to confirm their 3-dimensional extent and actual size. These were classified according to the American Association of Orthopaedic Surgeons (AAOS) system [5].

## Templating

Pre operative templating was performed as for primary total hip replacement in all patients. (In those having revision of the acetabulum and femoral components, a ceramic on ceramic couple was used in 15 cases and metal on ultra high molecular weight polyethylene in four cases). Where both components were revised, an acetabular cup 2-4 mm larger than the in situ cup was templated. In those patients in whom the acetabular component was retained, a matching modular cobalt chrome metal head was fixed to an uncemented stem.

## Operative Technique

All procedures were performed via a posterior approach. In cases where the acetabulum was preserved the femoral neck osteotomy was performed and the head was removed. Subsequent femoral preparation proceeded as for a primary total hip replacement. A straight, tapered reamer was inserted into the femoral canal followed by incremental rasps as appropriate. Once the stem was firmly seated, an appropriately sized large diameter cobalt chrome head with a modular neck (Smith and Nephew, Warwick, UK) was applied and reduction was performed.

In cases where both components were revised the femoral neck osteotomy was performed after dislocation of the joint. The in situ acetabular component was removed using the Explant device (Zimmer, Warsaw, Indiana) coupled to an adaptor device as previously describe [6]. Acetabular defects, if present, were packed with a combination of morsellised auto and allograft. Femoral revision proceeded as described above. All revision prostheses were uncemented. In cases of isolated femoral revisions, Synergy (n = 4) and Echelon (N = 1) stems (Smith & Nephew, Warwick, UK) were inserted to which a large diameter cobalt chrome head was applied. Where both components were revised, the metal on metal bearing was replaced by ceramic on ceramic components. A posterior capsular repair was performed in all cases. Sutures were placed into the capsule using the Mason-Allen technique [7] and attached to the posterior edge of the greater trochanter via drill holes.

## Post Operative Care

A drain was left deep to the fascia lata for 24 hours in all cases. All patients received 3 doses of prophylactic antiobiotics. Low molecular weight heparin, thromboembolic deterrent (TED) stockings and calf compression devices were used to decrease the risk of thromboembolic events. Patients who required bone graft for the acetabulum were mobilized partial weight bearing for the first four weeks while those not requiring graft were allowed to fully weight bear from day 1 post-operatively. Average duration of stay was 5 days (Range 4-7 days).

A course of physiotherapy was started 4-6 weeks post operatively in order to improve strength and flexibility of the abductors and hip flexors and facilitate gait retraining. Full activity was permitted from 3 months.

## Follow Up

**Figure 1:**
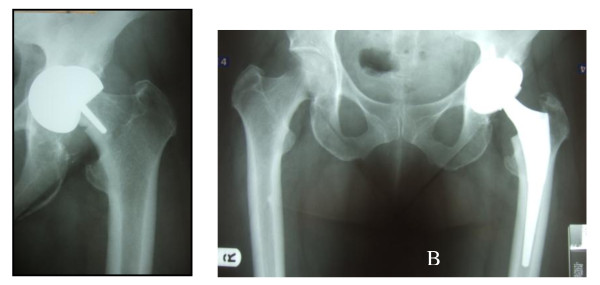
A- Preoperative X-ray study showing gross loosening of the socket with a femoral neck fracture; B- Post operative X-ray six months later.

Patients were routinely followed up at 4 weeks, 12 weeks, one year post operatively and at 3 yearly intervals afterward. Clinical and radiological evaluation were performed at each follow up visit. Stable fixation of both components was indicated by lack of radiolucent lines and lytic lesions and the presence of spot welds at the bone prosthesis interface as well as trabeculae extending to the uncemented stem [8] (Figure 1). Oxford, Harris and WOMAC hip scores were also recorded.

## Results

Twenty five patients were included in this study. There were 12 females and 13 males. The mean ages of the male and female cohorts were 62.2 (range 56-72 years) and 58.5 years (range 41 - 65 years) respectively. One patient was lost to follow up as she currently resides overseas but at 3 months she had returned to full function and had no pain. The average duration of follow up was 12.7 months (3-31). Eight patients were followed for a minimum of 24 months. The demographics of our patient cohort are illustrated in Table 2.

Indications for revision included pain localized to the groin (24%), pain not resolving after extended bouts of sport activity (8%), pain with clicking (8%), pain with an effusion (40%), dislocation (4%), femoral neck fracture secondary to a fall (8%) and infection (8%) (Table 3).

The average time to revision was 30.2 months (4 - 65 months) overall. Among female patients it was 26.4 months (7 - 60) and 34.4 months (4-65) in the male group (p = 0.27). The average femoral component size in the female group was 43 (38-50) compared to 49 (46-54) in the male patients (p = 0.0003, CI 3.27-8.93).

In cases where both components were revised, the average size of the explanted acetabular component was 50.7 mm (46 - 58 mm) compared to 54.6 mm (52 - 60) post revision. Intra operative findings were varied based on the diagnosis. All patients except those with femoral neck fractures had at least a small effusion. Three patients had black staining of the pseudo capsule and periarticular soft tissues suggesting deposition of metallic debris.

Effusions were charcoal coloured in 3 cases and cream coloured in one patient in the absence of infection. Cystic lesions were noted behind the acetabular component in 3 cases but the cup was grossly loose in only one of these. There was evidence of gross collapse of a segment of the femoral head (evidenced by softening of the bone at the margin of the prosthesis) in one patient.

**Figure 2:**
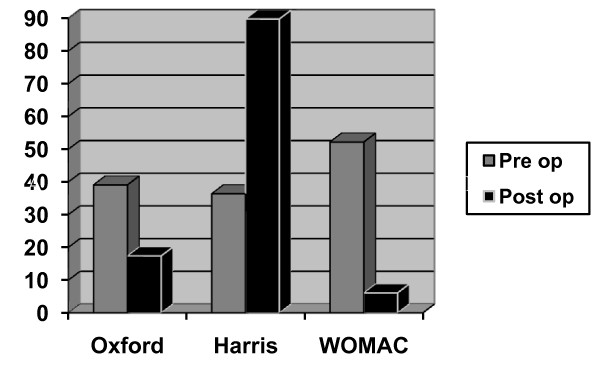
**Pre and post operative hip scores**.

Pre operative Oxford, Harris and WOMAC hip scores were 39.1, 36.4 and 52.2 respectively. Post operative scores were 17.4, 89.8 and 6.1 respectively p < 0.0001, p < 0.0001 and p < 0.0001 respectively (Figure 2). The greatest improvement was seen in the pain component of the Harris Hip Score with an average improvement of 35 units (79.5%) at the time of last follow up.

The average UCLA activity score increased from 3 to 8. Two patients had returned to extreme sports (though this was against our advice).

There were no cases of symptomatic leg length discrepancy, new infection or neurological complications post operatively. All patients except those with acetabular bone graft were allowed to fully weight bear day 1 post operatively. The average post operative length of stay was 5 days. All patients were satisfied with their outcome at their last follow up. Two patients were unable to access their shoelaces at 3 months post operatively. One patient had these complaints pre operatively while the other patient recovered his normal hip flexion after a prolonged course of physiotherapy. All patients have reported resolution of their pain post revision.

## Discussion

**Figure 3:**
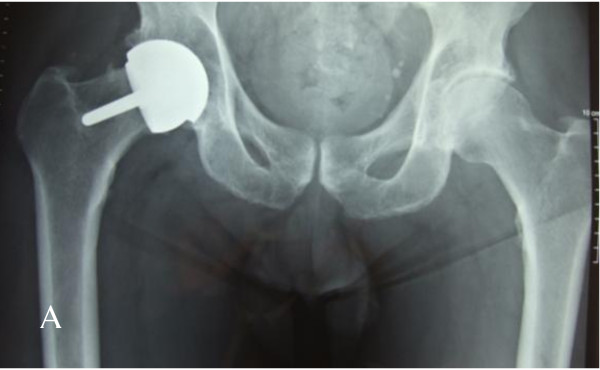
**Varus positioning of the femoral component**. This patient presented with progressive pain and inability to return to normal activity.

**Figure 4:**
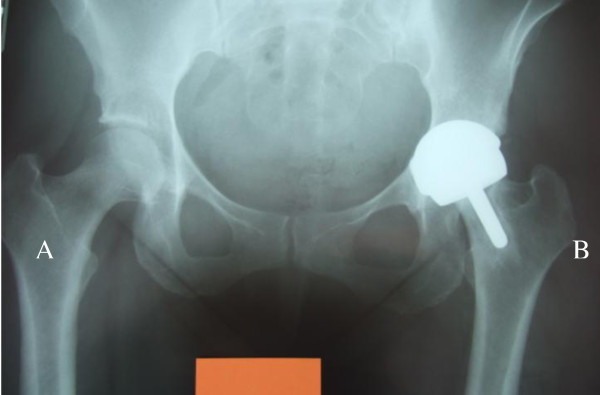
**Loosening of the femoral component (arrow shows the reactive lines around the loose stem)**.

**Figure 5:**
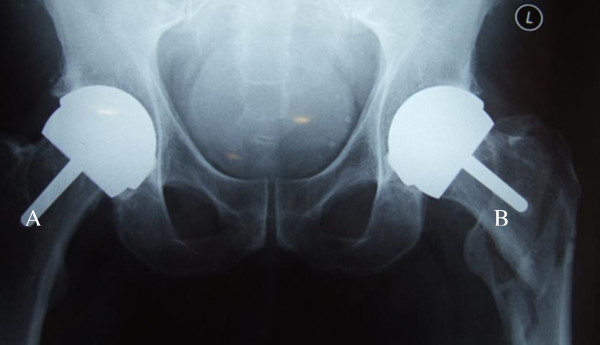
**A comminuted complex intertrochanteric fracture occurring due to the patient falling from his bicycle**. The acetabular component was retained in this case.

Metal on metal resurfacing arthroplasty has seen a rise in popularity over the last decade. Early results of contemporary resurfacing have shown success rates above 97.8% at a mean of 5 years in the young, active population [9]. Despite these good early results complications have been noted including femoral neck fractures [2] and (at present) ill defined hypersensitivity/immune reactions associated with the metal on metal bearings (Figures 1, 3, 4, 5). The aetiology of these reactions remains under investigation but is not fully characterized [3,10]. As a result we have chosen to adopt a descriptive classification of our findings until the spectrum of this pathology is fully known. Similarities have been found to the cohort described by Willert et al [3] including the early recurrence of pain similar to pre operative levels and the presence of an effusion or soft tissue swelling. Histological studies have revealed perivascular T and B lymphoctyte aggregation in the majority of these cases. All patients with this presentation were revised to ceramic on ceramic bearing couples. While it is not fully understood it would seem logical to avoid cobalt chrome components in the bearing couple when revising for this indication.

While it is too early to comment on the clinical outcomes of these prostheses, the main determinants of success in these patients are pain relief and return to their normal physical function. One of the main proposed benefits of hip resurfacing arthroplasty is an improved range of movement and stability of the large diameter bearing. These should theoretically confer increased range of movement theoretically allowing a higher level of function. Prior to having their primary procedures these patients were all involved in sporting activities (ranging from tennis to snowboarding) which were discontinued due to pain after their surgery. Up to the last follow up all patients had returned to their normal jobs, activities of daily living and sports. This corresponded to elimination of their pain and increased UCLA activity scores.

It has been stated that revision of a hip resurfacing to a total hip replacement is a relatively simple procedure. While there is no doubt that hip resurfacing conserves bone on the femoral side, it has been suggested that it removes more acetabular bone [11]. While preparation of the femoral component is similar to conventional hip arthroplasty, revision of the acetabular component can be a technically demanding procedure with the risk of acetabular bone loss. In this series only 1 of 20 cups was loose. The remainder had to be extracted from surrounding bone

There were no episodes of clinical deep vein thromboses (DVT's), leg length discrepancy or infection up to the time of last follow up. These early results compare favourably with similar reports for total hip replacements in young patients [12] and revision hip arthroplasty [13].

All patients were satisfied particularly by their pain relief. Average post operative Oxford, Harris and WOMAC hip scores were 17.4, 89.8 and 6.1 respectively. representing statistically significant improvements over pre operative scores (p < 0.0001 for each score). The group who had infected prostheses improved more slowly than their non infected counterparts but reported equal rates of satisfaction.

Two patients (1 female, 1 male) had infected prostheses requiring revision. Both patients presented with pain and effusions but no systemic symptoms. The infecting organisms were Staphylococcus Aureus Staphylococcus Epidermididis. These patients had normal looking wounds with no redness sinuses or discharge. Their erythrocyte sedimentation rates (ESR) were 48 and 27 and C- Reactive protein (CRP) levels were 96 and 56. Their White blood cell counts (WBC's) were less than 11 in both cases. No pus was discovered intraoperatively in these patients. They were both treated with one satge revisions and treated with six week courses of suitable antibiotics. The infection settled in both cases.

## Gender

**Figure 6:**
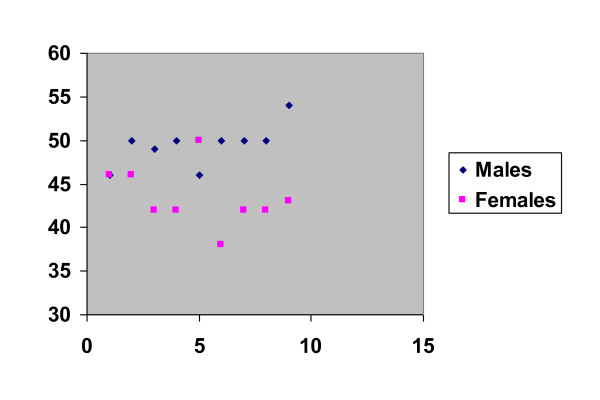
**Acetabular sizes based on gender**.

The ratio of male to female patients in our cohort is 1:1. The average age of females is 58.7 years (41 - 61)) and for males 61.5 years (51 - 72) reflecting higher failure rates in a younger female population. The reasons for revision based on gender are presented in Table 2. Four males (and no females) presented with symptoms relating to activity potentially reflecting increased activity in this group after hip resurfacing. Conversely the female cohort all presented with pain and effusions which were successfully treated by revision of the bearing couples. Osteolytic lesions behind the acetabular components were only noted in female patients. This is an interesting observation that is difficult to explain. It may be that it is a chance finding, though it may also suggest that hypersensitivity type reactions to metal on metal articulations are more common in females. Our cohort suggests show that female patients with smaller diameter bearing surfaces have higher failure rates. This has recently been reported in recently presented data from a series of over 1000 patients (Treacy, personal communication). The average sizes of femoral components based on gender is shown in Figure 6. It may be that the female gender is a surrogate marker for small component size and it may be that problems are more common with smaller size metal on metal bearings.

Excluding the patients with infection, hip scores were similar in male and female patients. Unexplained painful reactions often with an effusion seems to be a real phenomenon with a small proportion of metal on metal articulations. These symptoms can be so severe that revision is indicated. They seem to be more common in females. Revising them to a total hip replacement with non metal on metal bearings produces rapid early pain relief. This is associated with good objective outcome measures. Infection after hip resurfacing can be eradicated. Recovery, as with infection after total hip replacement is slower and ultimate hip scores are lower [14].

## Conclusion

Our paper shows that the short term outcome of revision of hip resurfacing to total hip replacement gives high patient satisfaction, good function and pain relief. Unexplained pain reactions seem to be more common in female patients with smaller diameter components while those who are revised due to infection progress more slowly. Techniques for maximal acetabular bone preservation have been described, particularly for the BHR component while conversion of a resurfacing femoral component is as bone conserving as a primary femoral stem [6].

While the complication rates in this group are encouraging compared to both primary and revision total hip replacement, caution should be used in drawing conclusions from this as the follow up period is relatively short and longer term results are necessary. It is also logical to assume that as the number of resurfacings increase, so will the number of revisions. This will provide larger series for study and also provide data based on component design.

## Competing interests

The authors declare that they have no competing interests.

## Authors' contributions

NS Reviewed the patients clinically, collected the data, organized and prepared the first draft of the paper. SMA identified the topic as a subject of current interest, reviewed the patients clinically and edited the written paper while JAS reviewed the radiographs, co-authored the discussion and results. All authors have approved the final manuscript.

## Informed Consent

Informed consent was obtained from each patient participating in this study. Permission was obtained for publishing the images used in this paper. A copy of this would be available for review by the Editor- in- Chief of this journal
